# GABA_A_ Alpha 2,3 Modulation Improves Select Phenotypes in a Mouse Model of Fragile X Syndrome

**DOI:** 10.3389/fpsyt.2021.678090

**Published:** 2021-05-21

**Authors:** Tori L. Schaefer, Amy A. Ashworth, Durgesh Tiwari, Madison P. Tomasek, Emma V. Parkins, Angela R. White, Andrew Snider, Matthew H. Davenport, Lindsay M. Grainger, Robert A. Becker, Chandler K. Robinson, Rishav Mukherjee, Michael T. Williams, Jay R. Gibson, Kimberly M. Huber, Christina Gross, Craig A. Erickson

**Affiliations:** ^1^Division of Psychiatry, Cincinnati Children's Hospital Medical Center, Cincinnati, OH, United States; ^2^Division of Neurology, Cincinnati Children's Hospital Medical Center, Cincinnati, OH, United States; ^3^Department of Pediatrics, University of Cincinnati College of Medicine, Cincinnati, OH, United States; ^4^Department of Neuroscience, University of Texas Southwestern Medical Center, Dallas, TX, United States

**Keywords:** Fragile X syndrome, FXS, FMRP, GABA, EEG, audiogenic seizures, novel object recognition, UP states

## Abstract

Fragile X syndrome (FXS) is the most common cause of inherited intellectual disability. FXS is caused by functional loss of the Fragile X Protein (FXP), also known as Fragile X Mental Retardation Protein (FMRP). In humans and animal models, loss of FXP leads to sensory hypersensitivity, increased susceptibility to seizures and cortical hyperactivity. Several components of the GABAergic system, the major inhibitory system in the brain, are dysregulated in FXS, and thus modulation of GABAergic transmission was suggested and tested as a treatment strategy. However, so far, clinical trials using broad spectrum GABA_A_ or GABA_B_ receptor-specific agonists have not yielded broad improvement of FXS phenotypes in humans. Here, we tested a more selective strategy in *Fmr1* knockout (KO) mice using the experimental drug BAER-101, which is a selective GABA_A_ α2/α3 agonist. Our results suggest that BAER-101 reduces hyperexcitability of cortical circuits, partially corrects increased frequency-specific baseline cortical EEG power, reduces susceptibility to audiogenic seizures and improves novel object memory. Other *Fmr1* KO-specific phenotypes were not improved by the drug, such as increased hippocampal dendritic spine density, open field activity and marble burying. Overall, this work shows that BAER-101 improves select phenotypes in *Fmr1* KO mice and encourages further studies into the efficacy of GABA_A_-receptor subunit-selective agonists for the treatment of FXS.

## Introduction

Fragile X syndrome (FXS) is the most common inherited form of intellectual disability and often associated with autism, anxiety, irritability, and attention deficit hyperactivity disorder (1). Although many potential small molecule candidates have been tested in clinical studies (2–4), there is currently no effective approved treatment of FXS.

FXS is caused by a trinucleotide (CGG) repeat expansion in the 5'UTR of the *FMR1* gene leading to its transcriptional silencing and loss of a single protein, the Fragile X Protein (FXP), also known as Fragile X Mental Retardation Protein (FMRP). FXP has many functions including, but not limited to, control of mRNA transport, translation, and stability, as well as binding to and regulating ion channels, and DNA repair (5). *Fmr1* knockout (KO) mice, the most frequently used animal model for FXS, display many phenotypes reminiscent of the human condition. They show, for example, hyperactivity, altered social preference, and impaired cognition, and are widely used to investigate pathological mechanisms of FXS and to preclinically test novel therapeutic strategies (6).

A particularly prominent characteristic of loss of FXP in humans and animal models is excessive brain activity. Studies in mice demonstrate that loss of functional FXP leads to altered and increased neuronal and circuit excitability, manifest, for example, in impaired synaptic plasticity, elevated neocortical activity and increased susceptibility to audiogenic seizures (7). Notably, humans with FXS are more prone to develop epilepsy than the general population (8), exhibit sensory hypersensitivity (9), and recent EEG studies have shown increased gamma power activity, altered neuronal synchronization and impaired connectivity in the neocortex of individuals with FXS (10, 11). Together, these findings suggest that altered circuit excitability is a disease-relevant and translational phenotype in FXS.

While the underlying molecular mechanisms of increased neuronal activity are not fully understood, there is substantial evidence for altered inhibitory transmission in FXS caused by changes in the γ-aminobutyric acid (GABA)ergic system (12–14). There are two classes of GABA receptors, the heteropentameric anion-permeable GABA type A (GABA_A_) receptor, which mediates fast inhibition (15), and the G-protein coupled GABA type B (GABA_B_) receptor, which mediates slow inhibition (16). GABA_A_ receptors consist of combinations of 19 different subunits (17). MRNA and protein of several of these receptor subunits are reduced in *Fmr1* KO mouse brain (18–21) and in humans (22). Moreover, expression of the rate-limiting GABA-synthesizing enzyme glutamic acid decarboxylase (GAD) is altered in *Fmr1* KO mice but the direction of the dysregulation is unclear and appears to depend on the brain region (18, 20, 23).

Based on these studies, GABA receptors were evaluated as potential therapeutic targets in FXS. These studies have mostly used receptor subunit-non-specific agonists of either GABA_A_ or GABA_B_ receptors (24, 25). Despite positive results using a GABA_B_ agonist, arbaclofen (R-Baclofen, STX209) the active racemic enantiomer of baclofen, in the FXS mouse model (26), a large Phase III trial in individuals with FXS with this drug did not meet the defined endpoint criteria (24). Similarly, ganaxolone, a GABA_A_ agonist did not lead to significant clinical improvements in humans (25) following positive preclinical reports (27). Interestingly, with both arbaclofen and ganaxolone, treatment-associated positive effects were noted in *post-hoc* subgroup analyses. This supports the therapeutic promise of targeting the GABAergic system in FXS, but also highlights the need to evaluate alternative, more selective GABA receptor modulators while working to build a priori justification of potentially targeting specific subgroups of persons with FXS with a specific GABA modulator.

The investigational drug BAER-101 (formerly known as AZD7325) is a selective GABA receptor modulator that activates the α2 and α3 subunits of the GABA_A_ receptor. This specific pharmacologic profile leads to potential potent anxiolytic actions without the common sedative impact of non-selective GABA_A_ agonists such as benzodiazepines (28). Here, we tested the effect of BAER-101 treatment on cortical circuit hyperexcitability, behavioral phenotypes, and memory in *Fmr1* KO mice. Our results suggest that low-dose BAER-101 may be beneficial to normalize circuit hyperexcitability and improve object recognition memory but did not improve anxiety- and repetitive behavior-related phenotypes in *Fmr1* KO mice. These studies encourage more detailed analyses in the mouse model and in humans to evaluate the potential benefits of BAER-101 and other GABA_A_ subunit-selective agonists in FXS.

## Materials and Methods

### Animals

*Fmr1* KO mouse breeding colonies (29, 30) were established in the Rodent Barrier Facility at Cincinnati Children's Hospital Medical Center (CCHMC) and at University of Texas Southwestern (UTSW). All protocols were approved by the Institutional Animal Care and Use Committees at CCHMC or UTSW. Mice for this study were housed under 14/10 (CCHMC) or 12/12 (UTSW) light/dark cycle at controlled temperature and humidity. Test subjects were generated from the mating of female *Fmr1*^+/−^ mice (30) with male WT mice on a C57BL/6J background. Male mice from these pairings were used as test subjects for juvenile audiogenic seizure tests and the adult behavior battery and extracellular signal-related kinase (ERK1/2) analysis. Mice used for neocortical slice recordings or EEG analysis and dendritic spine morphology were also generated by breeding female *Fmr1*^+/−^ mice to male WT mice on a C57BL/6J background, but the *Fmr1* KO strain was originally obtained from the *Jackson Laboratories* (Bar Harbor, ME) (29). While the two *Fmr1* KO mouse strains used here are slightly different in how they were generated, they both do not express FXP. All mice were genotyped on postnatal day (P) 10–28 by ear clip and weaned on P28. Male *Fmr1* KO and WT littermates were used for experiments and group housed throughout testing (with dam and litter of 2–4 per cage). Audiogenic seizures and UP state analyses were performed at P21 because *Fmr1* KO mice in C57BL/6J background are only susceptible to audiogenic seizures during early development and UP states neocortical slices are most robust at this age (31). Behavior, EEG, ERK1/2, and dendritic spine analyses were done in adult (2–4 months old) mice. Electrode and transmitter implanting as well as many of the behavioral assays are difficult or impossible during juvenile periods.

### Drug and Drug Dosing

BAER-101 (4-amino-8-(2-fluoro-6-methoxy-phenyl)-N-propyl-cinnoline-3-carboxamide hydrogen sulfate, formerly AZD7325) was obtained from AstraZeneca (Europe), and the vehicle Sulfobutylether-Beta-Cyclodextrin (SBECD) was supplied by AstraZeneca (US). SBECD is a pharmaceutical grade agent that is used as a solubilizing agent in drugs currently on the market in the US (Voriconazole). Mice were treated with either 3 mg/kg (high dose) or 1 mg/kg (low dose) of BAER-101 in 0.05% SBECD or vehicle (0.05% SBECD) in a volume of 5 ml/kg (3BAER, 1BAER, or VEH, respectively). Drug doses were based on IC50 values and recommendations communicated by AstraZeneca and mimicked the parallel human trial design that likewise included a high and a low dose group. Juvenile mice (3 weeks) were dosed by oral gavage one time, 30 min prior to the start of the audiogenic seizure test. For mice in the adult behavior battery, dosing commenced 10 days prior to the start of behavior analysis with treatment continuing during behavior testing. The 22 gauge gavage needles were 1.5” needles with 1.25 mm ball (Cadence Science catalog # 7920). A gavage dosing volume of 5 ml/kg was used to reduce the amount of SBECD exposure. On behavior testing days, dosing was staggered such that a period of 0.5 h would separate the dose and start of behavior for each mouse. For EEG analyses, mice implanted with cortical EEG electrodes and wireless transmitters were treated daily with 1 mg/kg BAER-101 or vehicle for 10 days either by oral gavage as described above or by providing the drug in a single-serve portion of peanut butter (32). The mice were observed to ensure they consumed the entire peanut butter, which usually took 1–2 min but always under 5 min. We used this method successfully in the past to deliver drugs (32). We changed drug administration methods from oral gavage to single-serve peanut butter to avoid disturbing the transmitter and electrode implants. We did not detect an effect of dosing method on EEG measurements in the limited number of mice tested (*data not shown*).

### Neocortical Slice Preparation and UP State Recordings

Slices from somatosensory barrel cortex (400 μm thick) were prepared using an angled block on a vibratome as described (31). Slices were incubated for 1 h at 32°C in artificial cerebrospinal fluid (ACSF: 126 mM NaCl, 3 mM KCl, 1.25 mM NaH_2_PO_4_, 26 mM NaHCO_3_, 2 mM MgCl_2_, 2 mM CaCl_2_, and 25 mM d-glucose), followed by perfusion with modified ACSF for 45 min (as above, but with 5 mM KC, 1 mM MgCl_2_, and 1 mM CaCl_2_). For drug treatment, 1 or 3 μM of BAER-101 was added during the entire 45 min in modified ACSF. Using 0.5 MΩ tungsten microelectrodes, spontaneously occurring UP states were recorded extracellularly from layer 4 of the primary somatosensory cortex for 10 min, amplified 10,000-fold, sampled at 2.5 kHz, and filtered on-line between 300 Hz and 5 kHz. UP state analysis was done with custom LabVIEW software. Briefly, recordings were offset to 0, rectified and a low-pass filter was set at 0.2 Hz cutoff frequency. The detection threshold was set at 4-fold of the root mean square noise. The beginning of an UP state was defined as events in which the amplitude remained above threshold for at least 200 ms. The end of the UP state was defined as a decrease of the amplitude below the threshold for >600 ms, whereas two events within 600 ms were defined as one single UP state. In the figure, *n* is the number of slices.

### Electrode Implanting

Electrode implanting and EEG recording were performed as described (33). Briefly, 6–8 week-old male *Fmr1* KO mice and littermate WT controls were implanted with single-channel wireless transmitters for EEG monitoring [TA11ETA-F10, Data Science International (DSI), St. Paul, MN] under isoflurane anesthesia. Mice were given analgesics (Carprofen) prior to and after the surgery, and surgical sites were disinfected with 2% Chlorhexidine. Dorsoventral coordinates were measured from bregma and two holes were drilled at AP = −2 mm, L = ± 4.0 mm. The two leads of the transmitter were inserted into the burr holes on top of the dura (~1 mm) and sealed with GLUture (Zoetis Inc., Kalamazoo, MI). The wireless transmitter was placed subcutaneously behind the neck. The assembly was secured with dental cement (Lang Dental, IL). After the cement had dried, the incision was closed using surgical sutures (Coviden, Dublin, Ireland) and sealed with GLUture. Mice were injected with 1 ml saline, placed on a heating pad, and monitored during recovery.

### EEG Recording and Analysis

After electrode implantation, mice were housed in individual cages placed on wireless receiver plates (RPC1; DSI). EEG data received from the telemetry system were recorded with DATAQUEST A.R.T software and sampled at 500 Hz, providing readouts for frequencies between 1 and 200 Hz (maximal sampling rate of the wireless transmitter TA11ETA-F10). Video was continuously recorded in parallel (Axis 221, Axis communication) and synchronized with the EEG signal. Daily treatment with 1 mg/kg BAER-101 or vehicle started 9–12 days after the surgery to allow for recovery and lasted for 10 days. Brains were collected 1–2 h after the last drug dose and processed for Golgi-Cox staining (*see below*). EEG data were analyzed with NeuroScore software (DSI) for 9 consecutive days starting at the day of the first dose and ending on the day of the ninth dose. A 5-min period of recording (free of excessive movement and grooming behavior to avoid artifacts) was selected from individual mice within 1–3 h of treatment (~12–2 pm each day). For EEG power analyses, the raw EEG signal was exported in 10 s epochs and subjected to Fast Fourier Transformation to generate power bands. The data were pooled from 10 s epochs for a total of 5 min duration per day and screened to remove artifacts. Averages from 5 min segments of 9 treatment days are shown. The EEG signal was split into power bands of the following frequencies: delta (δ, 0.5–4 Hz), theta (θ, 4–8 Hz), alpha (α, 8–12 Hz), sigma (Σ, 12–16 Hz), beta (β, 16–24 Hz) and gamma (γ, 24–80 Hz) (34). Power bands were compared between the *Fmr1* KO and WT BAER-101- and vehicle-treated mice. Separate analyses were performed after normalization of individual power bands to total power. A total of 27 mice were implanted with electrodes for this study, 2 died before or during treatment and 2 mice were used for a pilot study trying different drug doses and thus were removed from analysis. Of the remaining 23, 6 mice had to be removed because of EKG signal or highly noisy EEG (1 WT vehicle-treated, 1 WT BAER-101-treated, 2 *Fmr1* KO vehicle-treated and 2 *Fmr1* KO BAER-101-treated mice).

### Dendritic Spine Analysis

Dendritic spines from mice that underwent EEG analysis were visualized using the FD Rapid Golgi-Stain Kit from *FD Neurotechnologies, Inc*. (Columbia, MD) as we have done before (35). Briefly, brains were harvested 2–3 h after the last dose of BAER-101 or vehicle, Golgi impregnated, and then cut into 160 μm thick slices. The slices were stained following the manufacturer's protocol and imaged with a 60x oil objective using a Nikon inverted microscope. Secondary apical dendrites (50–150 μm length, ≥100 μm distant from the soma) of the hippocampal CA1 (bregma −1.8 to −2.2) were analyzed using ImageJ (*NIH*). Eleven to 15 dendrites from five to seven mice per condition were analyzed. Statistical analyses were based on dendrite number. Note that several mice from this cohort could not be used for EEG analyses as stated above but were used for dendritic spine analyses. All mice used for dendritic spine analysis had cortical surface electrodes implanted as described above and were treated with either 1 mg/kg BAER-101 or vehicle for 10 days.

### Juvenile Audiogenic Seizure Test

Male *Fmr1* KO and WT littermates were housed with their litter and dam and were treated via oral gavage with vehicle (VEH), 1 mg/kg BAER-101, or 3 mg/kg BAER-101 30 min prior to assessment. The audiogenic seizure test consisted of a 2 min priming tone (120 dB siren), which does not typically induce seizure behavior, followed by 1 min of silence and then a second tone (120 dB siren) lasting an additional 2 min (36). Each mouse was tested alone in a static mouse cage free of bedding. A Mugger Stopper Plus personal alarm was used to generate the tone and was placed on the filter cage lid with the speaker facing down into the cage. The battery was replaced often to ensure the sound intensity was always at maximum. During the second tone, behavioral response was scored as 0, 1, 2, 3, or 4 with 0 indicating no altered behavior, 1 indicating wild-running, 2 indicating clonic seizure (rapid limb flexion and extension), 3 indicating tonic seizure (static limb extension), and 4 indicating the most severe response of death (37). No seizure behavior was observed during the priming tone for this cohort of mice. Seizure severity during the second tone was calculated by using a mouse's most severe response number. Seizure severity was analyzed by the Exact Wilcoxon Rank sum test for non-parametric data. Treatment group (WT+VEH, WT+1BAER, WT+3BAER, KO+VEH, KO+1BAER, KO+3BAER) was used with exact probabilities calculated to determine pairwise group comparisons. These group comparisons were corrected using the false discovery rate (FDR) method.

### Adult Behavior Battery

Behavior was assessed during the light portion of the light/dark cycle and food and water were available *ad libitum* except during active behavior testing. Mice began testing on day 11 of treatment. To minimize the impact of stress during behavioral testing, mice were transported across the hallway to the Rodent Behavior Core and dosed with VEH, 1BAER, or 3BAER and allowed 30 min in the testing room to acclimate before behavior assessment. Elevated zero maze was the only exception in which mice were brought into the testing room one at a time just prior to being placed on the maze in order to get an accurate anxiety assessment. Mice were tested in only one paradigm per day, except for locomotor activity and marble burying, which were performed on the same day. Behavior was evaluated in the following order so that tests easily influenced by stress were completed early during the behavior battery: elevated zero maze (EZM), locomotor activity, marble burying, acoustic startle habituation, prepulse inhibition of startle, novel object recognition (NOR), rotarod. We also performed an adhesive removal assay and a pole descend assay as described (38, 39) before the rotarod assay but these experiments did not show genotype or drug effects, have not been shown to be altered in *Fmr1* KO mice before and are thus not reported in the manuscript. Apparatus surfaces were cleaned with Process NPD (Steris) before testing started and between mice. Sample sizes of treatment groups were as follows: WT vehicle: *n* = 23, WT 1BAER: *n* = 25, WT 3BAER: *n* = 24, *Fmr1* KO vehicle: *n* = 21, *Fmr1* KO 1BAER: *n* = 20, *Fmr1* KO 3BAER: *n* = 20. Mice were tested in 3 separate cohorts. Mice that were excluded from the analysis, if any (e.g., because they could not complete the task, were sick or died), are indicated under each assay below.

#### Elevated Zero Maze

The EZM was used to assess anxiety-like behavior as described with modification of the maze size (40). Briefly, mice were transported from the housing room to the testing room individually and placed on the apparatus. The experimenter exited the room immediately after placing the mouse in one of the closed quadrants of the apparatus. A camera mounted above the maze connected to a computer located outside the room was used to observe and score, in real-time, time in open quadrants and number of open arm entries (transitions during a single 5 min trial) (ODLog, Macropod Software). The test room was dimly lit (30 lux) to encourage exploration of the test environment. One *Fmr1* KO vehicle-treated mouse, 2 *Fmr1* KO 3BAER-treated mice and 2 WT 3BAER-treated mice were excluded from the EZM analysis.

#### Locomotor Activity

Locomotor activity was measured in infrared photocell activity chambers (41 × 41 cm; PAS Open Field, San Diego Instruments, San Diego, CA) for 1 h. Total Distance was recorded during 5 min intervals for a total of 60 min and analyzed with a 3-way ANOVA with repeated measures. Room lights were at full level (1,200 lux).

#### Marble Burying

Immediately after spontaneous locomotor activity assessment, mice were moved to an adjacent room and tested in a marble burying task. Through unpublished observations we found that assessing marble burying directly following locomotor activity elicits the most reliable degree of burying in control animals. Briefly, mice were placed in a standard rat cage containing 10 cm (depth) of woodchip bedding. Twenty marbles were evenly distributed on the surface of the bedding using a template in four rows of five. Mice were individually placed in the cage for 10 min and scored for the number of marbles at least 2/3 buried at the end of the testing session.

#### Novel Object Recognition

A solid black enclosure with dimensions 19.5 cm L × 40 cm W × 35 cm H was used to assess NOR. During the familiarization phase, mice were presented with two identical objects for a total of 5 min. Mice were returned to their cage and left undisturbed for 30 min. Next, mice were placed back in the enclosure with a novel object and one identical copy of the familiarization phase object. Pilot mice had no inherent preference for any of the objects used in this test (*data not shown*). The amount of time each mouse spent paying attention to the familiar and novel objects during the familiarization and test phases was recorded using OD Log (Macropod Software) for the 5 min duration of each phase. Time spent paying attention was recorded when the mouse was oriented toward the object with snout within 1 cm of the object or when forepaws were up against the object. Mice in these cohorts did not climb on top of the objects used for this test. The discrimination index (DI; novel object time—familiar object time/novel object time + familiar object time) was used to determine the degree of object memory. Dim lighting conditions (20 lux) were used to reduce anxiety and encourage object exploration during both phases. DI during the test phase was analyzed by 2-way ANOVA.

#### Acoustic Startle Habituation and Prepulse Inhibition (PPI)

Acoustic startle habituation and PPI were assessed in a sound-attenuating test chamber (SR-LAB apparatus; San Diego Instruments, San Diego, CA) as described with modifications (41). Mice were placed in an acrylic cylindrical holder that was mounted on a platform with a piezoelectric force transducer attached to the underside of the platform. For both habituation and PPI, a 5 min acclimation period preceded test trials. For habituation, each mouse received 50 repeated 20 ms 120 dB SPL mixed frequency sound bursts (1.5 ms rise time, analyzed in 5 blocks of 10 trials). Maximum startle amplitude for each trial (Vmax; measured in arbitrary units; a.u.) was analyzed by repeated measures 3-way ANOVA. For PPI, each animal received a 5 × 5 Latin square sequence of trials that were of 5 types: startle stimulus (SS) with no prepulse, 73 dB prepulse + SS, 77 dB prepulse + SS, or 82 dB prepulse + SS. The startle signal was a 20 ms 120 dB SPL mixed frequency sound burst (1.5 ms rise time). Prepulses preceded the startle-eliciting stimulus by 70 ms (onset to onset). The startle recording window was 100 ms. Background noise level was 70 dB. Each set of 25 trials was repeated 4 times for a total of 100 trials. The inter-trial interval averaged 14 s and varied randomly from 8 to 20 s. Vmax at each prepulse level was analyzed by 3-way mixed factor ANOVA with genotype and drug as between factors and PPI trial type as a within factor. Two *Fmr1* KO vehicle-treated mice, 1 WT 1BAER-treated mouse, and 1 WT 3BAER-treated mouse were excluded from PPI and startle analyses.

#### Rotarod

There were four trials each test day, and 2 days of testing. Mice were tested in bins, so all mice went through trial 1 before starting trial 2. Intertrial interval was a minimum of 10 min. The rod accelerated from 4 to 40 rpm over the first 180 s. The latency to fall from the rod, or a complete full rotation without walking, was recorded as the dependent variable and measured by the computer connected to the laptop. If a mouse did not fall off the rod, the full 300 s score was recorded. The investigator placed the mouse facing the back of the apparatus onto the stationary rod before the test was started.

### Extracellular Signal-Regulated Kinase (ERK1/2) Quantification

Following the adult behavior battery, mice continued to be treated for 3–5 additional days prior to sacrifice. Care was taken to minimize stress on the final day of treatment in which dosing occurred 30 min prior to sacrifice. Mice were removed from their cage, which was kept in their permanent housing room and transferred directly to necropsy one at a time. Decapitation occurred within 30 s from removal of the mice from the housing room. Brains were removed and maintained on ice. For ERK1/2 quantifications, the hippocampus was removed from one hemisphere and rapidly frozen onto a stainless-steel plate over dry ice. Once frozen, brain tissue was transferred to a microfuge tube and stored at −80C until assayed. For total protein determination, the hippocampus was homogenized in ice-cold RIPA buffer (500 μl and 100 μl, respectively), with the fresh addition of HALT phosphatase inhibitor cocktail (ThermoScientific) and protease inhibitor cocktail (Sigma) and assayed using the Pierce BCA Protein Assay Kit (ThermoScientific) according to manufacturer's instructions. Samples were diluted to 50 μg/ml for phosphorylated ERK1/2 (pERK1/2) and 2.5 μg/ml for ERK1/2 total prior to analysis. pERK1/2 and ERK1/2 total were analyzed by semiquantitative SimpleStep ELISAs (enzyme-linked immunosorbent assay; ABCAM; phosphoERK1/2 pT202/Y204, ab176640 and ERK1/2 Total, ab176641) according to manufacturer's instructions. Data were verified to fall within the linear range of the standard curve that was run on each plate and mean optical densities of duplicate samples was used for calculations. ERK1/2 total and the ratio of pERK1/2 over ERK1/2 total normalized to WT+VEH were analyzed by 2-way ANOVA with genotype (WT or *Fmr1* KO) and drug (VEH, 1 mg/kg, or 3 mg/kg BAER-101) as factors.

### Statistical Analysis

Behavioral data and ERK1/2 phosphorylation were analyzed using mixed linear factorial analysis of variance (ANOVA; Proc Mixed) with the exception of seizure severity score in which the Exact Wilcoxon Rank sum for non-parametric data was used (SAS v9.4, SAS Institute, Cary, NC). Significant main effects and interactions were followed-up with pairwise group comparisons using the False Discovery Rate (FDR) method to control for multiple comparisons (42). Specific details relating to between and within factors and repeated measures were briefly described above with specifics detailed in the Results. Data are shown as least squares (LS) mean ± standard error of the mean (SEM) for model consistency with the exception of seizure severity, EEG power, UP states, and dendritic spine density, in which ordinary means and SEM are shown. UP states and dendritic spine density were analyzed with 2-way ANOVA and EEG data were analyzed with ordinary 2-way ANOVA or mixed-effects analysis (restricted maximum likelihood, REML) because of missing values, followed by Tukey's *post-hoc* tests when significant interactions were observed using GraphPad Prism v8.1 or v9.0. Sample sizes are detailed in the Results section, and/or figure legends. All behavioral coding, slice analyses, dendritic spine counting, and molecular assays were performed by experimenters blind to genotype and treatment group. A *p* < 0.05 was considered significant and trends are reported at *p* < 0.1. Statistically significant differences in pairwise comparisons are reported in the figure and the figure legends. Details about sample sizes and statistical tests for each experiments are also provided in Supplementary Table 1.

## Results

Experimental mouse cohorts and the order in which assays were conducted are stated in the method section and differ from how they are discussed below.

### BAER-101 Reduces Brain Hyperexcitability in *Fmr1* KO Mice

Individuals with FXS are hyperactive, hypersensitive to auditory and visual stimuli, and have increased gamma frequency band power in resting state dense-array EEGs (10, 11). This cortical hyperexcitability is replicated in the mouse model on brain network and behavioral levels. Because BAER-101, as a GABA_A_ agonist, is expected to enhance inhibitory signaling, we assessed if the drug reduces hyperexcitable network activity in *Fmr1* KO mice by testing its effect on neocortical UP states, cortical EEG abnormalities, and audiogenic seizures.

#### BAER-101 Normalizes Prolonged Up States

**Figure 1:**
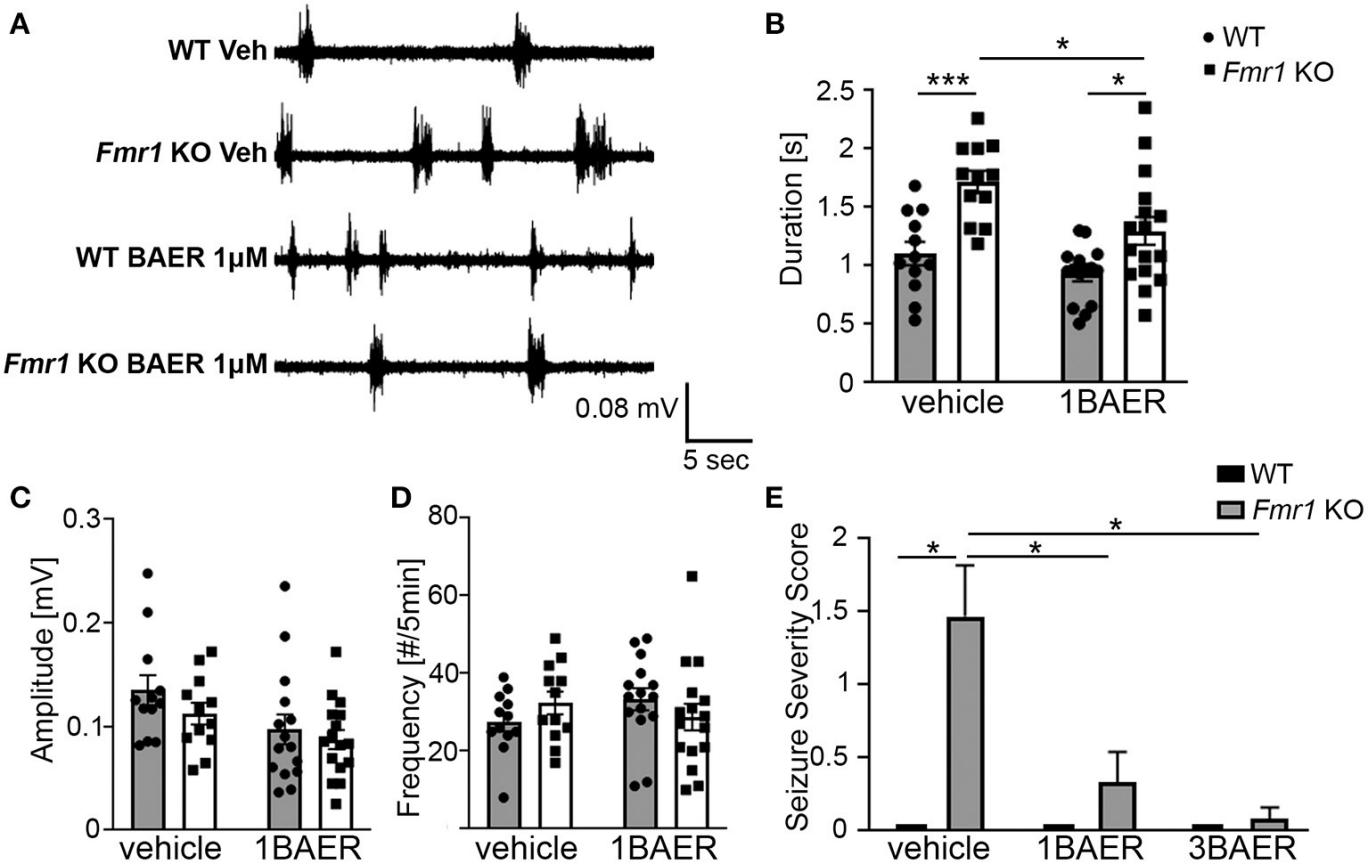
BAER-101 reduces neocortical hyperexcitability and seizures in *Fmr1* KO mice. **(A**–**D)** Increased duration of spontaneous UP states in neocortical slices from *Fmr1* KO mice is reduced to WT levels by bath application of 1 μM BAER-101. **(A)** Representative extracellular multi-unit recordings from layer IV of acute neocortical slices prepared from WT or *Fmr1* KO mice and preincubated for 1–1.5 h in either BAER-101 (1 μM) or vehicle (0.03% DMSO). **(B)** Increased duration of UP states in *Fmr1* KO slices is reduced by BAER-101 [2-way ANOVA with Sidak's multiple comparison, *p*(genotype) < 0.0001, *p*(drug) = 0.004, *p*(interaction) = 0.22]; ***p < 0.001; *p < 0.05. **(C)** Amplitude of UP states was reduced by BEAR-101 but not affected by genotype and no interaction was detected [2-way ANOVA, *p*(genotype) = 0.2, *p(drug) = 0.015, *p*(interaction) = 0.61]. **(D)** UP state frequency was not affected by genotype or treatment [2-way ANOVA, *p*(genotype) = 0.95, *p*(drug) = 0.71, *p*(interaction) = 0.12]. Sample sizes for UP states were as follows (slices): WT vehicle: *n* = 12, WT 1BAER: *n* = 15, *Fmr1* KO vehicle: *n* = 12, *Fmr1* KO 1BAER: *n* = 16. **(E)** Audiogenic seizures are significantly reduced by administration of 1 mg/kg and 3 mg/kg BAER-101 (1BAER and 3BAER, respectively), 30 min before testing (Kruskal-Wallis test, *p* = 0.0001; Wilcoxon rank pair-wise comparisons with FDR correction: *p < 0.01). Quantification of seizure scores suggests a dose-dependent effect with stronger reduction with 3 mg/kg BAER-101. WT vehicle: *n* = 12, WT 1BAER: *n* = 10, WT 3BAER: *n* = 11, *Fmr1* KO vehicle: *n* = 17, *Fmr1* KO 1BAER: *n* = 15, *Fmr1* KO 3BAER: *n* = 13.

**Figure 2:**
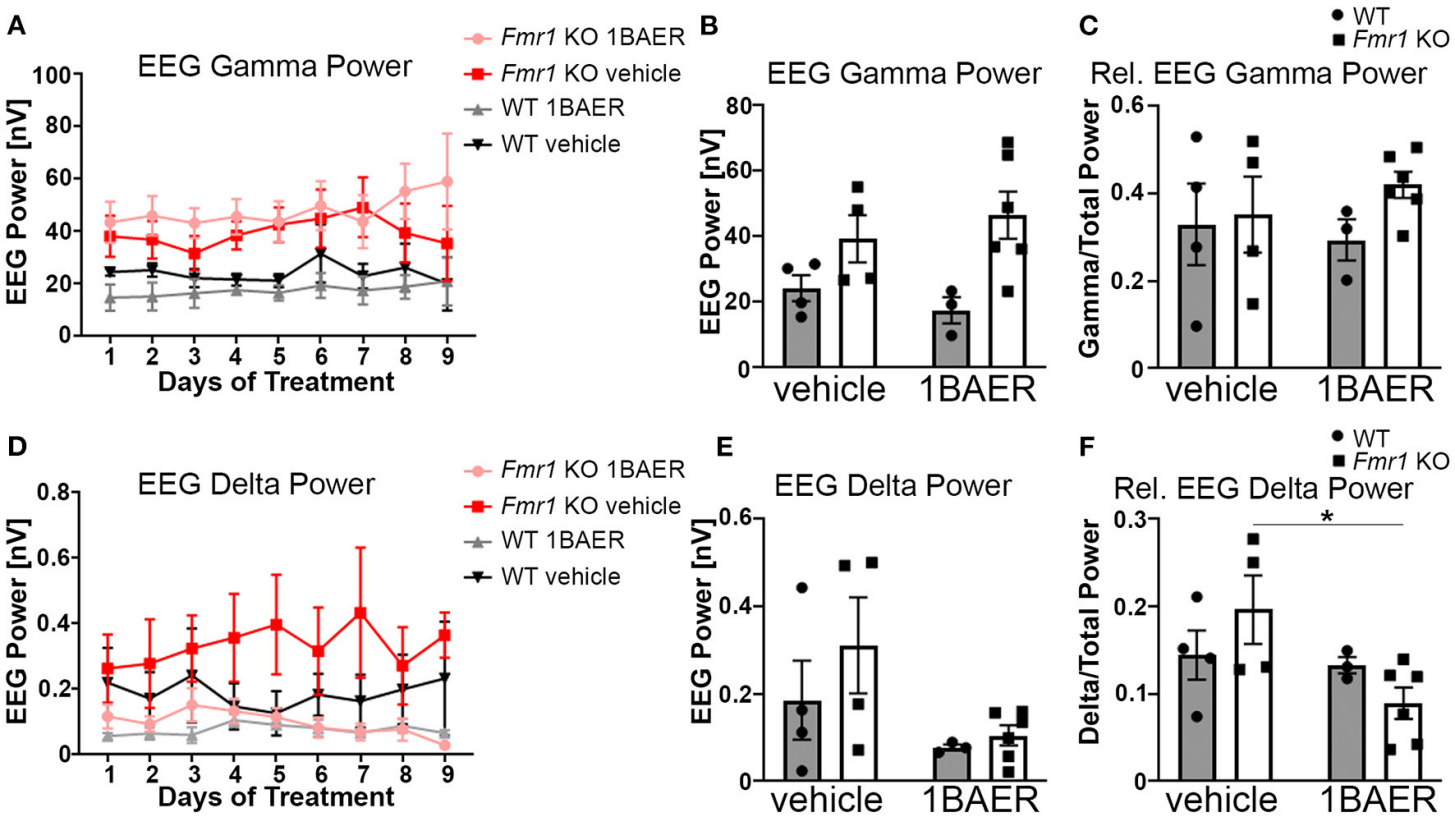
Low-dose BAER-101 reduces increased delta EEG power but not gamma EEG power in *Fmr1* KO mice. **(A–C)** Gamma EEG power is increased in *Fmr1* KO mice but not affected by daily treatment with 1 mg/kg BAER-101 (1BAER) over 9 days. The effect of drug or genotype on absolute gamma EEG power does not change during the 9 day treatment period [**A**, separated by day, mixed-effects analysis, *p*(genotype) = 0.007, *p*(day) = 0.30, *p*(day x treatment) = 0.72; *p*(day x genotype) = 0.99, *p*(day x treatment x genotype) = 0.80; no other significant effects; **(B)**, 9-day average, 2-way ANOVA, *p*(treatment) = 0.98, *p*(genotype) = 0.007, *p*(interaction) = 0.33]. Relative gamma power (normalized to the EEG power across all frequency bands) shows on average non-significantly increased power in *Fmr1* KO mice compared with WT [**C**, 9-day average, 2-way ANOVA, *p*(treatment) = 0.81, *p*(genotype) = 0.29, *p*(interaction) = 0.45]. **(D–F)** Delta EEG power is reduced by daily treatment with 1 mg/kg BAER-101 over 9 days. Similarly as for gamma EEG power, the effect of drug or genotype on absolute delta power does not change over the 9-day period [**D**, separated by day, mixed-effects analysis, *p*(treatment) = 0.047, *p*(day) = 0.58, *p*(day x treatment) > 0.99; *p*(day x genotype) = 0.40, *p*(day x treatment x genotype) = 0.31; no other significant effects; **(E)** 9-day average, 2-way ANOVA, *p*(treatment) = 0.045; *p*(genotype) = 0.30, *p*(interaction) = 0.50]. Relative delta power in *Fmr1* KO is significantly reduced by 1 mg/kg BAER-101 whereas no effect on WT was observed [**F**, 2-way ANOVA with Sidak's *post-hoc* test, *p*(treatment) = 0.046; *p*(genotype) = 0.87, *p*(interaction) = 0.10; *p = 0.04]. WT vehicle: *n* = 4; *Fmr1* KO vehicle: *n* = 4; WT 1BAER: *n* = 3; *Fmr1* KO 1BAER: *n* = 6. EEG power was analyzed during a 5 min period within 1–3 h after drug dosing (~12–2 pm each day). Analysis of other waveforms is shown in Supplementary Figure 1.

UP states, a type of persistent activity state of local neocortical circuits, are prolonged in cortical slices of *Fmr1* KO mice, which reflects local circuit hyperexcitability (31, 43). Pre-treatment of *Fmr1* KO cortical slices with 1 μM BAER-101 significantly reduced the duration of UP states in *Fmr1* KO mice compared with vehicle-treated *Fmr1* KO slices [Figures 1A,B, 2-way ANOVA, ^*^p(genotype) < 0.0001, ^*^p(drug) = 0.004, *p*(interaction) = 0.22; Sidak's multiple comparisons *post-hoc* tests, ^***^p < 0.001; ^*^p < 0.05]. UP state amplitude (Figure 1C) was not significantly different between WT and *Fmr1* KO slices, but reduced by 1 μM BAER-101 independently of genotype [2-way ANOVA, *p*(genotype) = 0.2, ^*^p(drug) = 0.015, *p*(interaction) = 0.61]. UP state frequency (Figure 1D) was not significantly different between WT and *Fmr1* KO slices and not affected by 1 μM BAER-101 [2-way ANOVA, *p*(genotype) = 0.95, *p*(drug) = 0.71, *p*(interaction) = 0.12]. Incubation with 3 μM BAER-101 reduced UP state duration even further but also reduced UP state amplitude below WT levels (*data not shown*) and was therefore not further analyzed. These results suggest that selective GABA_A_ modulation with BAER-101 reduces neocortical circuit hyperactivity in FXS.

#### Susceptibility to Audiogenic Seizures Is Reduced by Baer-101 in A Dose-Dependent Manner

Juvenile *Fmr1* KO mice are susceptible to audiogenic induced seizures whereas WT mice (C57BL/6 background) of all ages and adult *Fmr1* KO mice are mostly resistant. This increased susceptibility to audiogenic seizures is reminiscent of sensory hypersensitivity in individuals with FXS (44). Analysis of the seizure severity score in response to a 120 dB sound stimulus in 3-week old *Fmr1* KO mice and WT littermates 30 min after one dose of vehicle, 1 mg/kg BAER-101, or 3 mg/kg BAER-101 showed a significant effect of treatment (Kruskal-Wallis statistic: 24.86, ^*^p = 0.0001) (Figure 1E). Exact probabilities were computed to determine pairwise comparisons corrected using FDR and revealed significant increases in seizure severity score in the vehicle-treated KO mice compared with all other groups (^*^p < 0.01 for all comparisons). These data support the previously reported susceptibility to audiogenic seizures in *Fmr1* KO mice and indicate a significant treatment effect in both the low and high dose BAER-101-treated *Fmr1* KO mice (1 and 3 mg/kg BAER-101). The higher dose of BAER-101 on average decreased the seizure score even further compared with 1 mg/kg but there was no statistically significant difference between the two doses (Figure 1E). These results suggest that selective GABA_A_-modulation reduces sensory hyperexcitability in *Fmr1* KO mice.

#### Low-Dose BAER-101 Reduces Increased Delta Frequency Band Power but Does Not Correct Increased Gamma Frequency Band Power in *Fmr1* KO Mice

Resting gamma EEG power is increased in *Fmr1* KO mice and in humans with FXS (11, 45), suggesting a translationally relevant phenotype. To test the effect of BAER-101 on resting-state brain activity in the absence of FXP, we performed cortical surface EEG recordings from the auditory cortex of *Fmr1* KO mice and WT littermates paired with video recordings for 9 consecutive days during which mice were treated daily with either 1 mg/kg BAER-101 or vehicle. EEG power was analyzed during a 5-min period each day 1–3 h after dosing. The analysis period was chosen based on the video recordings to ensure that mice were sitting still to avoid artifacts caused by grooming or movement. These analyses confirmed that resting gamma power is increased in the auditory cortex of *Fmr1* KO mice [Figure 2A, mixed-effects 3-way ANOVA, ^*^p(genotype) = 0.007]; but no significant effects of day (*p* = 0.30), treatment (*p* = 0.93), or any interactions (all *p* > 0.3) were observed. Because we did not detect significant effects of day or any significant interactions of day with genotype, drug, or both, we compared the 9-day average with very similar results [Figure 2B, 2-way ANOVA, ^*^p(genotype) = 0.007; *p*(treatment) = 0.98, *p*(interaction) = 0.33]. To account for variability in signal intensity between mice and to better mimic human EEG analyses we also calculated relative power by normalizing gamma power to total power across all frequencies. While overall trends were the same, no significant effect of genotype on relative gamma power was observed [Figure 2C, 2-way ANOVA, *p*(genotype) = 0.3; *p*(treatment) = 0.81; *p*(interaction) = 0.45]. A previous study showed that apart from gamma power, delta power is also increased in the auditory cortex of *Fmr1* KO mice (45). In line with these findings, we observed on average increased delta power in *Fmr1* KO mice compared with WT littermates (Figures 2D–F). Analyses per day (Figure 2D) and averaged across the 9-day recording period (Figure 2E) showed significant effects of treatment, reducing delta power independently of genotype, but no other effects [**2D**, 3-way ANOVA, all *p* > 0.3, except for ^*^p(treatment) = 0.047; **2E**, 2-way ANOVA, *p*(genotype) = 0.30, *p*(interaction) = 0.50, ^*^p(treatment) = 0.045]. This suggests a selective effect of 1 mg/kg BAER-101 on delta but not gamma EEG power. Of note, there was a trend toward a significant interaction between genotype and treatment for relative delta power [Figure 2F, 2-way ANOVA *p*(interaction) = 0.10] and a significant reduction of relative delta EEG power in BAER-101 treated *Fmr1* KO mice compared with vehicle-treated *Fmr1* KO mice but not for WT mice, suggesting that the drug differentially affects relative delta power depending on genotype (Figure 2F, Tukey *post-hoc* test, ^*^p = 0.04). As reported previously (45), none of the other frequency bands' power (relative or absolute) was significantly different in the auditory cortex of *Fmr1* KO mice compared with WT littermates (theta, alpha, sigma) with the exception of absolute (but not relative) beta power, which was overall increased in *Fmr1* KO mice [^*^p(genotype) = 0.03] with no effect of treatment [*p*(treatment) = 0.38] (Supplementary Figure 1). Overall, these studies support previous findings of altered cortical activity in *Fmr1* KO mice and suggest that 1 mg/kg BAER-101 frequency band-specifically reduces increased resting brain EEG power in the absence of FXP.

### Short-Term Treatment of Adult Mice With Low-Dose BAER-101 Does Not Correct Increased Dendritic Spine Density in *Fmr1* KO Mice

**Figure 3:**
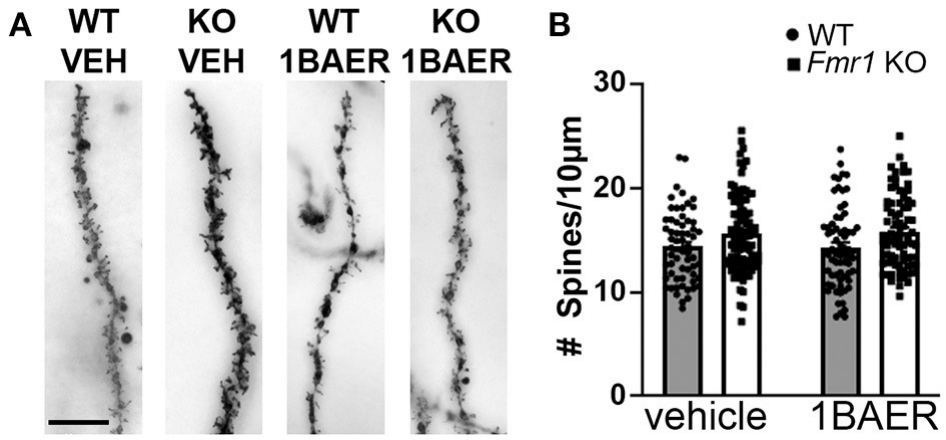
Increased dendritic spine density in *Fmr1* KO hippocampus is not affected by short-term treatment with low-dose BAER-101. Dendritic spine density on apical CA1 hippocampal dendrites from *Fmr1* KO and WT mice after 10 days of daily treatment with vehicle or 1 mg/kg BAER-101 (1BAER) was visualized by Golgi staining and quantified by manual counting using ImageJ. Representative images are shown in **(A)** quantifications in **(B)**. Dendritic spine density is increased in *Fmr1* KO mice but not changed by BAER-101 treatment [2-way ANOVA, *p*(treatment) = 0.87; *p*(genotype) = 0.0013, *p*(interaction) = 0.72]. Scale bar is 10 μm. WT vehicle: *n* = 61 dendrites from 5 mice (12–13 dendrites each); *Fmr1* KO vehicle: *n* = 94 dendrites from 7 mice (13–16 dendrites each); WT 1BAER: *n* = 67 dendrites from 5 mice (13–14 dendrites each); *Fmr1* KO 1BAER: *n* = 78 dendrites from 6 mice (11–15 dendrites each).

Dendritic spine density is increased and dendritic spine morphology altered in humans with FXS and in *Fmr1* KO mice, which may contribute to the observed brain circuit defects discussed above (46). We tested whether daily dosing with 1 mg/kg BAER-101 for 10 days normalizes dendritic spine density in the hippocampal CA1 region. We confirmed increased dendritic spine density on secondary apical dendrites of *Fmr1* KO mice compared with WT littermates, but did not detect an effect of treatment [Figure 3, 2-way ANOVA, ^*^p(genotype) = 0.001; *p*(treatment) = 0.90; *p*(interaction) = 0.72]. We speculate that longer dosing, and thus longer-term modulation of GABA_A_, is necessary to correct dendritic spine density in *Fmr1* KO mice.

### BAER-101 Alters Select FXS-Specific Behavioral Phenotypes

To evaluate the effects of BAER-101 treatment on behavioral phenotypes in *Fmr1* KO mice and WT littermates, we treated adult mice daily with 1 mg/kg or 3 mg/kg BAER-101 or vehicle. Daily treatment begun 10 days before the start of behavioral assays and continued throughout the behavioral assessments. The order of behaviors is stated in the methods and differed from how they are presented here. The completion of the behavioral battery lasted between 2 and 3 weeks for all cohorts, during which time daily treatment continued.

#### BAER-101 Increases Locomotor Activity in the Open Field

**Figure 4:**
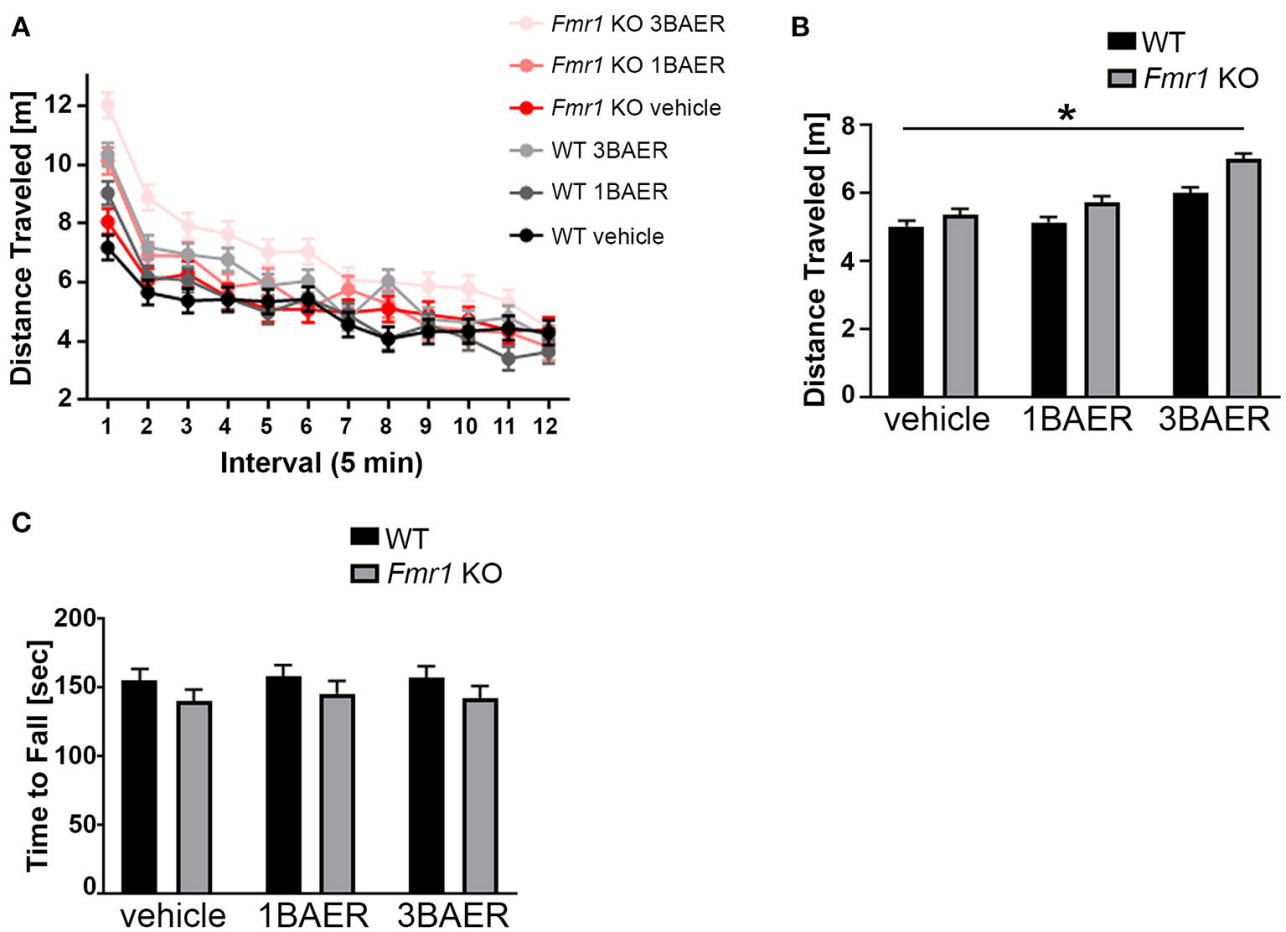
BAER-101 increases motor activity regardless of genotype but does not affect motor coordination. **(A,B)** Distance traveled in an open field is overall increased in *Fmr1* KO mice compared with WT littermates and further increased by BAER-101 treatment [**A**, 3-way ANOVA, *p*(genotype) < 0.0001, *p*(treatment) < 0.0001, *p*(interval) < 0.0001, *p*(treatment x interval) < 0.0001, *p*(treatment x genotype) = 0.16, *p*(treatment x interval x genotype) = 0.79]. FDR-corrected pairwise comparisons of data collapsed over time show significant differences for WT vehicle compared with *Fmr1* KO 3 mg/kg BAER-101 (**B**, *p = 0.0015). **(C)** *Fmr1* KO mice fell off the rotarod faster than their WT littermates but both genotypes improved over time. No effect of low- or high-dose BAER-101 treatment was detected [3-way ANOVA, *p*(genotype) = 0.044, *p*(treatment) = 0.87, *p*(day) < 0.0001, *p*(genotype x treatment) = 0.98, *p*(genotype x day) = 0.70, *p*(treatment x day) = 0.33, *p*(genotype x treatment x day) = 0.71]. Figure in **(C)** shows data collapsed over 2 days. WT vehicle: *n* = 23, WT 1BAER: *n* = 25, WT 3BAER: *n* = 24, *Fmr1* KO vehicle: *n* = 21, *Fmr1* KO 1BAER: *n* = 20, *Fmr1* KO 3BAER: *n* = 20.

To assess if GABA_A_ modulation by BAER-101 affects locomotor activity and coordination, we performed open field analyses and rotarod assays (Figure 4). Overall, we detected no or only genotype-unspecific effects of BAER-101 on these measures.

##### High-Dose BAER-101 Increases Locomotor Activity

Activity analysis in an open field, an overall indication of an animal's activity level, is sensitive to sedative drugs (including GABA modulators) or those inducing stereotypy or catatonia and is especially useful in better interpreting other tasks that depend on the overall activity of the animal. We therefore tested all mice in the open field for 60 min, separated into twelve 5-min intervals for analysis. In summary, all mice were more active in the beginning of the testing session, *Fmr1* KO mice were overall more active [as we and others reported before (47)], and BAER-101 treatment increased activity further (Figure 4A, 3-way repeated measures ANOVA, main effects of interval, genotype, and treatment: ^*^p < 0.0001). Apart from a significant treatment x interval interaction (^*^p < 0.0001) there were no other significant interactions, indicating no genotype-specific treatment effect. Although vehicle-treated *Fmr1* KO mice on average traveled further than vehicle-treated WT littermates, there was only a significant difference between *Fmr1* KO mice treated with 3 mg/kg BAER-101 and vehicle-treated WT littermates in pairwise comparisons [Figure 4B, FDR-corrected pairwise comparisons on data collapsed over time, *p*(wt/veh-ko/veh) = 0.12; ^*^p = 0.002]. These data suggest that high-dose BAER-101 causes elevations in activity levels when *Fmr1* KO mice are placed in a novel environment.

##### BAER-101 Does Not Affect Motor Coordination in Rotarod Experiments

To assess the effect of *Fmr1* gene deletion and BAER-101 on motor coordination, we used a rotarod test. In this test mice have to walk and balance on a horizontal rod that rotates around its own axis. The assay is performed twice, on two consecutive days, and the time to fall is used as a measure for motor coordination (48). As expected, all mice improved from day 1 (129.34 s +/– 4.35 s) today 2 (171.78 s +/– 4.35 s) [3-way repeated measures ANOVA; *p*(day) < 0.0001]. We observed that, overall, *Fmr1* KO mice fell off the rod earlier than WT mice [^*^p(genotype) = 0.044]; however there was no main effect of drug and no interaction effects indicating that BAER-101 did not affect motor coordination (Figure 4C, average data across both days are shown).

### BAER-101 Alters Anxiety-Related Behavior in Mice

**Figure 5:**
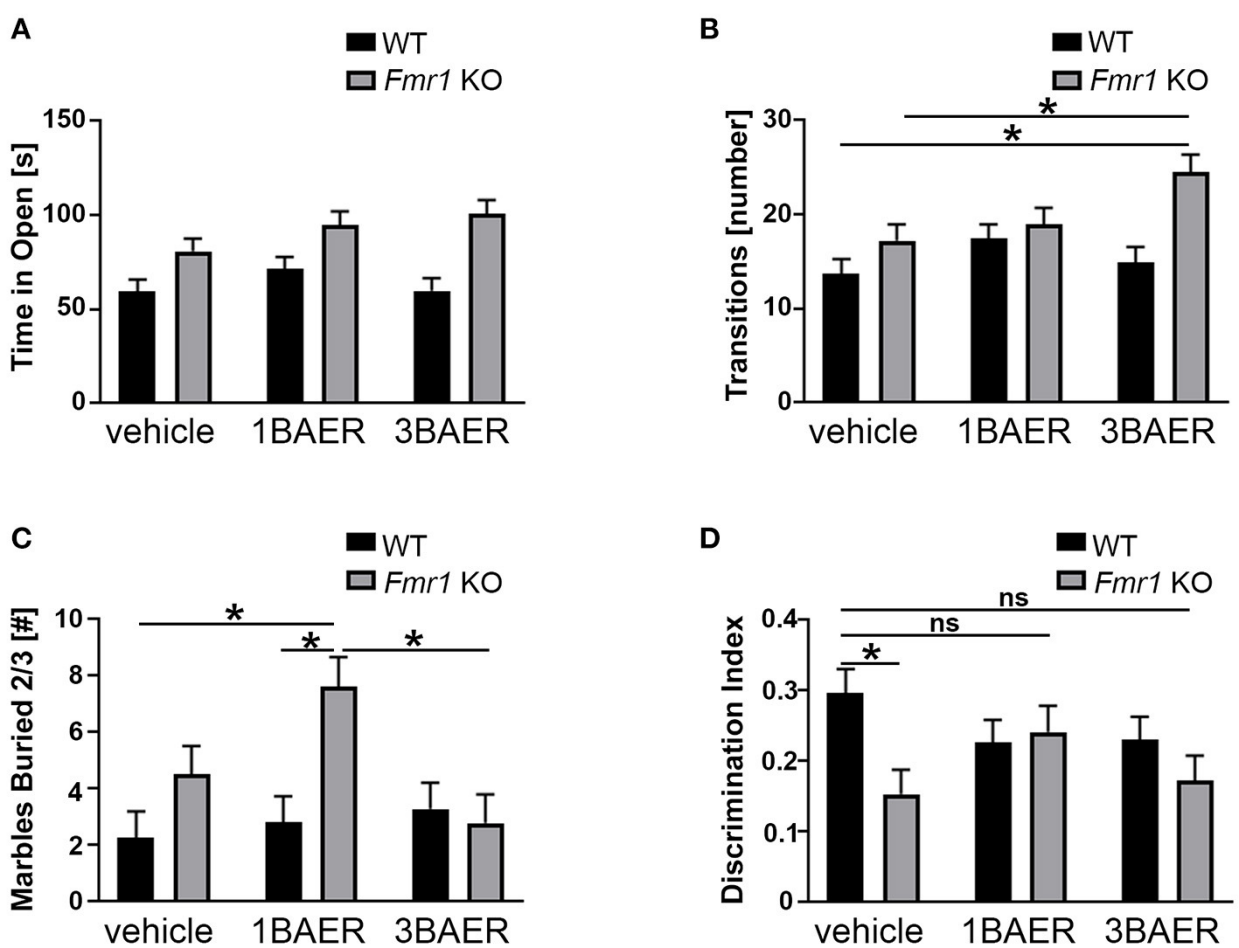
BAER-101 may worsen anxiety-related and repetitive behaviors but improve memory in *Fmr1* KO mice. **(A,B)** *Fmr1* KO mice spent more time in the open **(A)** and made more transitions **(B)** than WT littermates in the elevated zero maze; BAER-101 does not affect time in the open but 3 mg/kg BAER-101 increases the number of transitions between open and closed compartments [**A**, 2-way ANOVA, *p*(genotype) < 0.0001, *p*(treatment) = 0.13, *p*(interaction) = 0.32; **B**, 2-way ANOVA with FDR-corrected pairwise comparisons, *p*(genotype) = 0.0007, *p*(treatment) = 0.047, *p*(interaction) = 0.056, *p(wt/veh-ko/3BAER) = 0.0015, *p(ko/veh-ko/3BAER) = 0.019]. **(A,B)** WT vehicle: *n* = 23, WT 1BAER: *n* = 25, WT 3BAER: *n* = 23, *Fmr1* KO vehicle: *n* = 19, *Fmr1* KO 1BAER: *n* = 20, *Fmr1* KO 3BAER: *n* = 20. **(C)** Increased marble burying behavior in *Fmr1* KO mice is enhanced by 1 mg/kg BAER-101 [2-way ANOVA with FDR-corrected pairwise comparisons, *p*(genotype) = 0.008, *p*(treatment) = 0.066, *p*(interaction) = 0.03, *p(wt/veh-ko/1BAER) = 0.005, *p(wt/1BAER-ko/1BAER) = 0.008, *p(ko/1BAER-ko/3BAER) = 0.008). Number of marbles buried by two thirds or more after 10 min is shown. **(D)** 1 mg/kg BAER-101 may improve impaired novel object recognition memory in *Fmr1* KO mice [2-way ANOVA with FDR-corrected pairwise comparisons, *p*(genotype) = 0.028, *p*(treatment) = 0.62, *p*(interaction) = 0.073, *p(wt/veh-ko/veh) = 0.040, all other pairwise comparison not significant]. Shown is the discrimination index DI [(time with the novel object—-time with familiar object)/(time with the novel object + time with the familiar object)]. **(C,D)** WT vehicle: *n* = 23, WT 1BAER: *n* = 25, WT 3BAER: *n* = 24, *Fmr1* KO vehicle: *n* = 21, *Fmr1* KO 1BAER: *n* = 20, *Fmr1* KO 3BAER: *n* = 20. *indicates a significant difference, *ns* indicates not significant.

We used the EZM assay to assess anxiety behavior in vehicle- and BAER-101-treated *Fmr1* KO and WT mice during a 5-min test. Time in the open arm and number of transitions between the open and closed arm (= open arm entries) were measured (Figures 5A,B). We detected significant increases for time in open and open arm entries in the *Fmr1* KO mice compared with WT mice [2-way ANOVAs, ^*^p(genotype) < 0.0001 and 0.0007, respectively]. Neither low- nor high-dose BAER-101 affected time in open [*p*(treatment) = 0.13, *p*(interaction) = 0.32] but BAER-101 treatment increased the open arm entries [^*^p(treatment) = 0.047, *p*(interaction) = 0.056]. This effect was driven by the high-dose (3 mg/kg) BAER-101 treatment group: *Fmr1* KO mice made significantly more transitions after 3 mg/kg BAER-101 compared with vehicle-treated *Fmr1* KO mice and vehicle-treated WT littermates (FDR-corrected pairwise comparisons, ^*^p = 0.019 and 0.002, respectively). We speculate that increased activity in *Fmr1* KO mice after treatment with high-dose BAER-101, as seen in the open field test, influenced the *Fmr1* KO phenotype in EZM behavior.

### BAER-101 Does Not Reverse Increased Repetitive Behavior in *Fmr1* KO Mice

Marble burying is used to gauge repetitive behavior, and *Fmr1* KO mice exhibit increased burying compared with WT mice indicating enhanced repetitive behavior (49). We therefore tested the effect of BAER-101 treatment on this phenotype. After 10 min, *Fmr1* KO mice in general buried more marbles than their WT littermates, as expected, and 1 mg/kg but not 3 mg/kg BAER-101 increased the number of buried marbles in *Fmr1* KO mice further [Figure 5C; 2-way ANOVA, ^*^p(genotype) = 0.008; ^*^p(interaction) = 0.031; FDR-corrected pairwise comparison ^*^p(ko/veh-ko/1BAER) = 0.008, ^*^p(wt/1BAER-ko/1BAER) = 0.008, *p*(ko/veh-ko/3BAER) = 0.42].

### BAER-101 May Improve Impaired Memory in *Fmr1* KO Mice in the Novel Object Recognition Assay

Impaired novel object recognition in *Fmr1* KO mice was observed by others (50) and may reflect cognitive deficits associated with FXS. To assess the effect of BAER-101 on this phenotype we determined the time mice spent with a familiar and a novel object in a short-term object recognition test (51). A discrimination index (DI) was used to quantify novel object memory. All groups spent more time with the novel object as indicated by a DI greater than zero, but vehicle-treated *Fmr1* KO mice performed worse than WT littermates [Figure 5D, 2-way ANOVA with FDR-corrected pairwise comparisons, ^*^p(gene) = 0.028; *p*(interaction) = 0.073; ^*^p = 0.004]. By contrast, *Fmr1* KO mice treated with either 1 mg/kg or 3 mg/kg BAER-101 were not significantly different from vehicle-treated WT littermates (*p* = 0.87 and *p* = 0.11, respectively), and 1 mg/kg BAER-101-treated *Fmr1* KO mice showed on average increased (i.e., improved) DI compared with vehicle-treated *Fmr1* KO mice (62.1+/−1.8 vs. 57.7+/−1.7). These results confirm previous studies showing that *Fmr1* KO mice are impaired in short-term object recognition memory and suggest that low-dose BAER-101 may improve this phenotype.

### BAER-101 Does Not Alter Sensory Gating or ERK1/2 Activation

Prepulse inhibition (PPI) is a test of startle reactivity and sensorimotor gating and is impaired in young males with FXS, but enhanced in adult male mice (52). Although the reasons for these discrepancies between species are unknown, these previous studies suggest that both mice and people lacking FXP exhibit aberrant sensorimotor gating (52, 53). We therefore assessed how BAER-101 affects PPI in *Fmr1* KO mice. To acclimate the mice to the chamber and sound used for PPI, acoustic startle habituation was performed. All mice habituated to the sound as expected [2-way ANOVA, ^*^p(burst block) < 0.0001], and there was no effect of either 1 or 3 mg/kg BAER-101 treatment (*p* = 0.44) or genotype (*p* = 0.28), and no significant interaction (*p* = 0.41). After the acclimatization phase, PPI was assessed for each mouse at each of the prepulse trial types (PPI0, PPI73, PPI77, PPI82, numbers are indicating dB for each trial). These experiments did not replicate the previously described increase in PPI in *Fmr1* KO mice and no effect of treatment was observed [3-way mixed effect ANOVA, *p*(genotype) = 0.89, *p*(drug) = 0.91, ^*^p(type) < 0.0001, *p*(genotype x drug) = 0.32, *p*(genotype x type) = 0.13, *p*(drug x type) = 0.97, *p*(genotype x drug x type) = 0.09]. The results of these experiments should be interpreted with caution as the PPI response of the mice was very weak and all prepulses essentially elicited the same reduction in startle response.

To evaluate if BAER-101 corrects molecular defects, such as altered cellular signaling, in the FXS mouse model, we used phospho-ERK1/2- and ERK1/2-specific ELISAs to quantify ERK1/2 phosphorylation in hippocampal lysates from the mice after they underwent the behavioral testing. In a previous study we showed that ERK phosphorylation is increased in the hippocampus of *Fmr1* KO mice (47); however, here, we only detected a trend of increased pERK/ERK in *Fmr1* KO hippocampus and no significant interaction or drug effects [*n* = 8–10 per group, 2-way ANOVA, *p*(genotype) = 0.09, *p*(treatment) = 0.23, *p*(interaction) = 0.73]. Neither low- nor high-dose BAER-101 significantly changed ERK1/2 phosphorylation in the mice (Supplementary Figure 2). We assume that the relative absence of this molecular phenotype could have been caused by the four or more weeks of treatment, behavior testing, and daily handling. Indeed, while many studies demonstrated that neurotransmitter receptor-dependent cellular signaling is altered in FXS, there are partially contradictory findings regarding the steady-state activity of certain pathways, which are mostly attributed to differences in mouse and tissue handling (54). Stress can have significant effects on gene expression (55) most likely altering the molecular pathways that are changed in *Fmr1* KO mice (e.g., ERK1/2, PI3K/mTOR, and GSK3α/β signaling), which could confound molecular analyses. We therefore abstained from analyzing other FXS-associated molecular defects in these mice.

## Discussion

Novel disease mechanism-targeted treatments for FXS are urgently needed. A hallmark of FXS is an overall hyperexcitable brain network, which may be partially caused by impaired inhibition through GABAergic signaling. Here, we tested a novel therapeutic strategy in a mouse model of FXS targeting a subset of GABA_A_ receptors. GABAergic signaling has long been suggested as treatment target in FXS, but so far, preclinical and clinical studies mostly targeted broad spectrum GABA_A_ and GABA_B_ receptors, with mixed successes. Our strategy is novel since we used an investigational drug, BAER-101 that selectively targets only two (out of 19 possible) GABA_A_ receptor subunits, α2 and α3. Our studies suggest that BAER-101 can reverse neuronal circuit hyperactivity and improve memory in FXS but is ineffective in correcting hyperactivity and repetitive behavior in an FXS mouse model.

Several of our results indicate that BAER-101 at least partially corrects altered inhibitory neuronal transmission in *Fmr1* KO mice. First, in an *in vitro* approach, we showed that bath application of BAER-101 normalizes prolonged duration of UP states, suggesting that neocortical hyperactivity is normalized with the treatment. Second, we showed that BAER-101 significantly reduces the susceptibility to audiogenic seizures in a dose-dependent manner. This suggests that hypersensitivity to sensory stimuli, which is also seen in humans with FXS and most likely reflects a hyperactive and hyperexcitable neuronal network, is corrected by BAER-101. Third, we showed that BAER-101 normalizes enhanced delta EEG power. Enhanced EEG power of select frequency bands can be observed in mice and humans with no or very low levels of FXP, is believed to reflect neocortical hyperexcitability and may serve as a translational biomarker (10, 11, 45). Delta EEG power is associated with cognitive processing and believed to suppress networks not involved in a certain task (56). Notably, the correction of enhanced delta power was associated with improvement in novel object recognition memory, suggesting that correction of EEG power alterations in FXS is a valuable treatment goal.

In contrast to delta EEG power, increased gamma EEG was not rescued by BAER-101. Apart from being an agonist for the α2 and α3 GABA_A_ receptor subunits, BAER-101 also has neutral antagonistic action toward α1 (AstraZeneca, *personal communication*), which could influence its effect on EEG power bands. Moreover, we evaluated EEG power only during a 9-day treatment period. We speculate that longer treatment is necessary for a more comprehensive rescue of EEG power deficits, as well as for improvement of the dendritic spine phenotype which was likewise not rescued by this treatment paradigm. Lastly, we limited the EEG and dendrite studies to the low-dose BAER-101 condition. This decision was made following analysis of the behavioral data that indicated potentially enhanced efficacy of low- vs. high-dose drug in the KO mouse; however, we cannot exclude that the higher dose BAER-101 (3 mg/kg) would have been more effective in correcting alterations in gamma EEG power and dendritic spine density in *Fmr1* KO mice. This is particularly relevant, as gamma EEG oscillations are important for sensory processing (57), and audiogenic seizures, a form of hyperresponsivity to sensory stimuli, were most effectively reduced with 3 mg/kg BAER-101. It is thus conceivable that a higher dose of BAER-101 would have been necessary to rescue increased gamma EEG power. A limitation of the EEG analyses in the current study is the low number of animals tested. Future studies with larger sample numbers are needed to draw definitive conclusions.

A benefit of the selective action of BAER-101 on α2 and α3 GABA_A_ receptor subunits is its lack of sedative effects, which usually limits the clinical usability of broad GABA_A_ agonists such as benzodiazepines in persons with developmental disability. In fact, we observed *increased* activity in BAER-101-treated mice. Mice also spent more time in the open in the EMZ, suggesting reduced anxiety, which is in line with previous reports that α2 and α3 subunits mediate the anxiolytic effects of unselective GABA receptor agonists (58, 59), but is not consistent with normalization of *Fmr1* KO mouse behavior in this assay thus potentially limiting the face validity of this behavior test. A limitation of our study is that we cannot exclude that the drug-induced increase in activity altered other behavioral phenotypes tested. Current and future clinical trials will have to carefully monitor the effects of BAER-101 on hyperactivity-related symptoms in individuals with FXS.

The increased activity in BAER-101-treated mice may have contributed to the appearance of potentially worsening of the repetitive behavior in the marble burying assay in mice treated with low-dose BAER-101 and could have masked a potential beneficial effect on these repetitive/perseverative behaviors often associated with autism. A recent study supports this notion by showing that GABA_A_, but not GABA_B_ receptor agonism reduces marble burying behavior in WT mice (60), corroborating a potential beneficial effect of BAER-101 on perseverative behaviors. Interestingly, no increase in marble burying was observed with the higher dose of 3 mg/kg BAER-101. Instead, the 3 mg/kg BAER-101 appeared to normalize the phenotype (although no statistical significance was reached). This suggests that higher doses are needed to rescue repetitive behavior. We speculate that the opposing effects of low- and high-dose BAER-101 on marble burying may be due to the α1 antagonistic effect of BAER-101 that could have different influences on this phenotype depending on drug dose. In the future, it will be important to assess other autistic-like phenotypes in BAER-101 treated *Fmr1* KO mice to further evaluate its potential to ameliorate autism disorders in FXS. Additionally, different doses of BAER-101 could be evaluated to minimize hyperactivity-inducing actions of the drug.

It is worth noting that the *Fmr1* KO phenotypic representation is subtle and dependent on genetic background and environment, leading to contradictory phenotypes between different laboratories. In this study, we were not able to consistently replicate previously reported changes in PPI or ERK1/2 phosphorylation in *Fmr1* KO mice, and the effect of BAER-101 treatment on these phenotypes could thus not be determined reliably. Moreover, some of the effects we observed were subtle and our assays may have not been sufficiently powered or sensitive enough to detect significant changes. Future studies with larger sample size may be necessary to further analyze the effects of BAER-101 on these phenotypes. Nevertheless, the promising results in brain hyperactivity (UP states, audiogenic seizures, certain EEG frequency bands) and novel object recognition justify further evaluation in preclinical studies and clinical trials.

Based on our results in the mouse model, we predict that low-dose BAER-101 may have a beneficial effect on circuit excitability, sensory hypersensitivity, and cognitive function in FXS. The GABA_B_ receptor subunit-selective agonist arbaclofen did not meet social end point criteria in a large clinical trial (24). It will thus be interesting to test in follow-up studies how BAER-101 affects phenotypes of sociability [e.g., social novelty or social preference (61)] in *Fmr1* KO mice. It will be important in first-in-human studies to confirm whether low-dose BAER-101 shows a preferential positive clinical effect consistent with this preclinical report.

There are many different GABA_A_ receptor subunits expressed in the brain that can exist in a variety of different receptor combinations. The proportion of α receptor subunits within GABA_A_ receptors depends on the brain region and can affect receptor function (62). Our studies suggest that GABA_A_ agonists selective for specific α subunits improve certain phenotypes in *Fmr1* KO mice. The mRNA levels of several GABA_A_ subunits are downregulated in *Fmr1* KO mouse cortex (α1, α3, α4, β1, β2, γ1, and γ2) (20). While we did not analyze GABA_A_ subunit expression in our cohort of mice, this previous observation provides justification for assessing agonists specific to one or more of these GABA_A_ subunits as disease-targeted treatment in FXS. In selecting which subunit(s) to target it is important to consider previous studies suggesting that some of the GABA_A_ subunits shown to be altered in the *Fmr1* KO mouse model have functions that make them less attractive drug targets in FXS. For example, both α4 and β1 subunits play a role in alcohol intake and binge drinking (63, 64), increasing the risk of addiction. Moreover, the α1 subunit mediates sedative effects but not anxiolytic effects of benzodiazepines (65). By contrast, the α2/3-selective agonist BAER-101 was shown to be anxiolytic but not sedative making it a preferred candidate as novel therapeutic strategy. Nevertheless, future studies are necessary to evaluate which GABA receptor subunits (or what combinations thereof) are the most beneficial to modulate for the treatment of FXS.

## Data Availability Statement

The raw data supporting the conclusions of this article will be made available by the authors, without undue reservation.

## Ethics Statement

The animal study was reviewed and approved by Institutional Animal Care and Use Committees of Cincinnati Children's Hospital Medical Center and University of Texas Southwestern.

## Author Contributions

TS conceived, designed, analyzed, coordinated the behavior studies, wrote a first draft of the results, and methods section of the manuscript. AA, CR, and MD performed the ERK1/2 experiments. AA carried out the behavior studies along with MD, RB, and LG. CG, DT, and AW conceived and designed the EEG and dendritic spine studies. DT performed the EEG surgeries and recordings. AW and CG collected tissue samples thereafter. DT, AS, RM, and CG analyzed the EEG experiments. EP performed the Golgi staining, imaging, and dendritic spine analysis. MT, JG, and KH performed and analyzed the slice electrophysiology experiments. MW analyzed some of the behavior data. CG wrote the full draft of the manuscript. CE participated in the conceptualization of this study specifically, was responsible for bringing the BAER-101 molecule into the FXS field of study and edited the manuscript. All authors read, edited, and/or approved the final manuscript.

## Conflict of Interest

TS and CE are inventors on a method of use patent describing BAER-101 use in FXS that is held by the Cincinnati Children's Hospital Research Foundation (CCRF). CCRF has licensed this intellectual property to Baergic Bio. Neither AstraZeneca nor Baergic Bio participated in study design or in any aspects of the preparation, editing, or the content of this manuscript. AstraZeneca did provide free of cost the API (AZD7325/BAER-101) under a material transfer agreement with the CCRF. The remaining authors declare that the research was conducted in the absence of any commercial or financial relationships that could be construed as a potential conflict of interest.
