# Supplementary material for: GABAA Alpha 2,3 Modulation Improves Select Phenotypes in a Mouse Model of Fragile X Syndrome
Source: Front Psychiatry. 2021 May 21;12:678090. doi: 10.3389/fpsyt.2021.678090 (PMC8175776; doi:10.3389/fpsyt.2021.678090)
Supplement: Supplementary file 1 [file Data_Sheet_1.pdf]

Supplementary Material

GABA$_A$ alpha 2,3 modulation improves select phenotypes in a mouse model of Fragile X Syndrome

Tori L. Schaefer[1], Amy A. Ashworth[1]; Durgesh Tiwari[2,3]; Madison P. Tomasek[4], Emma V. Parkins[2]; Angela R. White[2]; Andrew Snider[2]; Matthew H. Davenport[1], Lindsay M. Grainger[1], Robert A. Becker[1], Chandler K. Robinson[1], Rishav Mukherjee[2], Michael T Williams[2,3], Jay R. Gibson[4], Kimberly M. Huber[4], Christina Gross[2,3]✝, Craig A. Erickson[1,3]✝

[1]Division of Psychiatry, Cincinnati Children's Hospital Medical Center, Cincinnati, OH, 45229 USA
[2]Division of Neurology, Cincinnati Children's Hospital Medical Center, Cincinnati, OH, 45229 USA
[3]Department of Pediatrics, University of Cincinnati College of Medicine, Cincinnati, OH, 45267
[4]Department of Neuroscience, University of Texas Southwestern Medical Center, Dallas, TX 75390 USA

Supplementary Figure 1

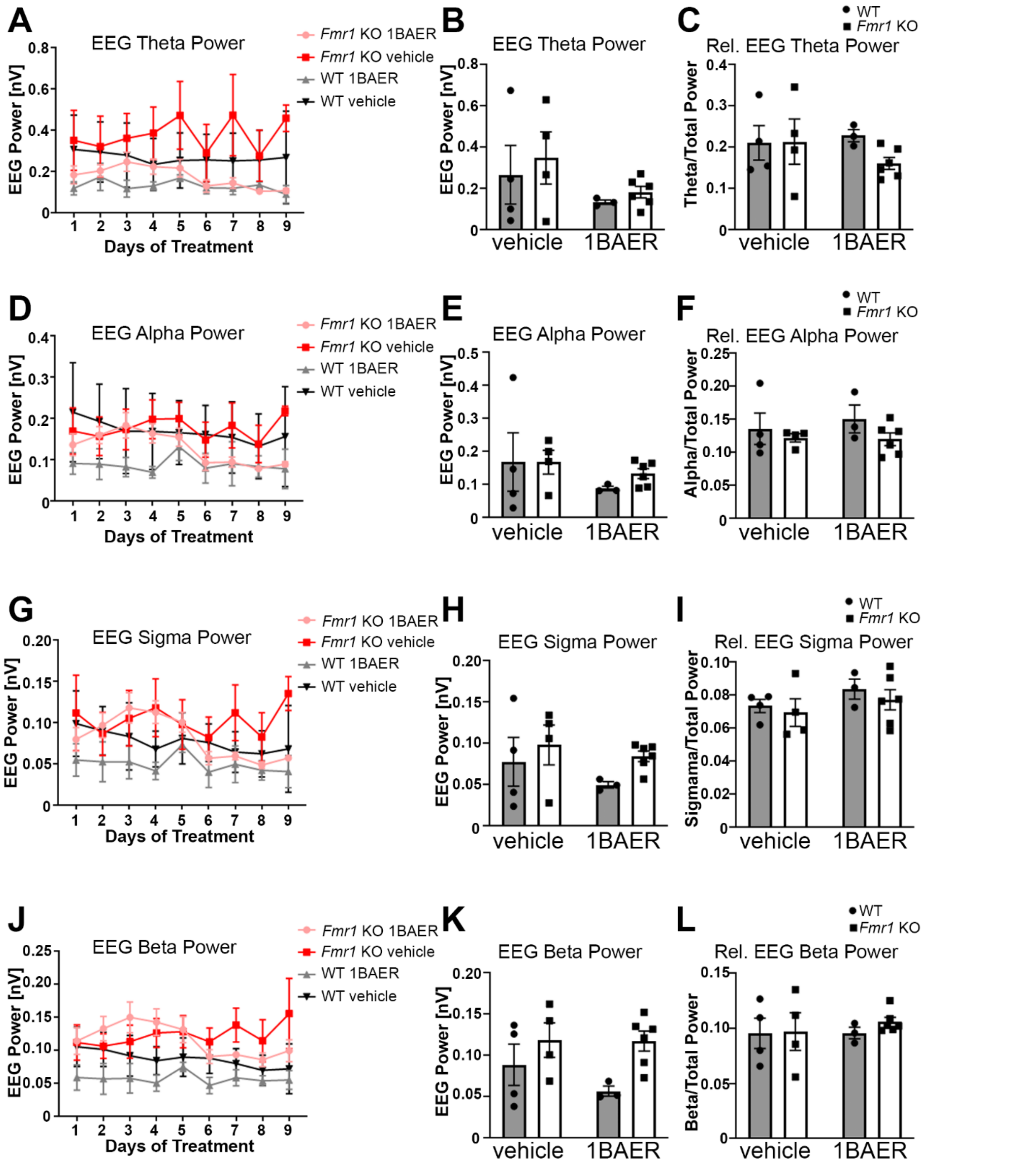

Supplementary Figure 1. Low-dose BAER-101 does not significantly affect theta, alpha, beta, or sigma EEG power in mice. (A-C) Theta EEG power is not significantly affected by *Fmr1* genotype or by daily treatment with 1 mg/kg BAER-101 (1BAER) over 9 days. The effect of drug or genotype on absolute theta EEG power does not change during the 9-day treatment period (**A**, separated by day, mixed-effects analysis, no significant effects; **B**, 9-day average, 2-way ANOVA, p(treatment)=0.14, p(genotype)=0.50, p(interaction)=0.86). Relative theta power (normalized to the

EEG power across all frequency bands) is likewise not affected by genotype or treatment (**C**, 9-day average, 2-way ANOVA, p(treatment)=0.63, p(genotype)=0.38, p(interaction)=0.34). **(D-F)** Alpha EEG power is not significantly affected by *Fmr1* genotype or by daily treatment with 1 mg/kg BAER-101 over 9 days. The effect of drug or genotype on absolute alpha EEG power does not change during the 9-day treatment period (**D**, separated by day, mixed-effects analysis, no significant effects; **E**, 9-day average, 2-way ANOVA, p(treatment)=0.25, p(genotype)=0.65, p(interaction)=0.64). Relative alpha power (normalized to the EEG power across all frequency bands) is likewise not affected by genotype or treatment (**F**, 9-day average, 2-way ANOVA, p(treatment)=0.69, p(genotype)=0.18, p(interaction)=0.59). **(G-I)** Sigma EEG power is not significantly affected by *Fmr1* genotype or by daily treatment with 1mg/kg BAER-101 over 9 days. The effect of drug or genotype on absolute sigma EEG power does not change during the 9 day treatment period (**G**, separated by day, mixed-effects analysis, no significant effects; **H**, 9-day average, 2-way ANOVA, p(treatment)=0.29, p(genotype)=0.17, p(interaction)=0.71). Relative sigma power (normalized to the EEG power across all frequency bands) is likewise not affected by genotype or treatment (**I**, 9-day average, 2-way ANOVA, p(treatment)=0.21, p(genotype)=0.46, p(interaction)=0.86). **(J-L)** Absolute, but not relative beta EEG power is significantly increased in *Fmr1* KO but not affected by daily treatment with 1 mg/kg BAER-101 over 9 days. The effect of drug or genotype on absolute beta EEG power does not change during the 9 day treatment period (**J**, separated by day, mixed-effects analysis, p(genotype)=0.028, no other significant effects; **K**, 9-day average, 2-way ANOVA, p(treatment)=0.38, p(genotype)=0.028, p(interaction)=0.42). Relative beta power (normalized to the EEG power across all frequency bands) is not affected by genotype or treatment (**L**, 9-day average, 2-way ANOVA, p(treatment)=0.69, p(genotype)=0.61, p(interaction)=0.70). WT vehicle: n=4; *Fmr1* KO vehicle: n=4; WT 1BAER: n=3; *Fmr1* KO 1BAER: n=6. EEG power was analyzed during a 5-min period within 1-3 hours after drug dosing (~12-2 pm each day).

Supplementary Figure 2

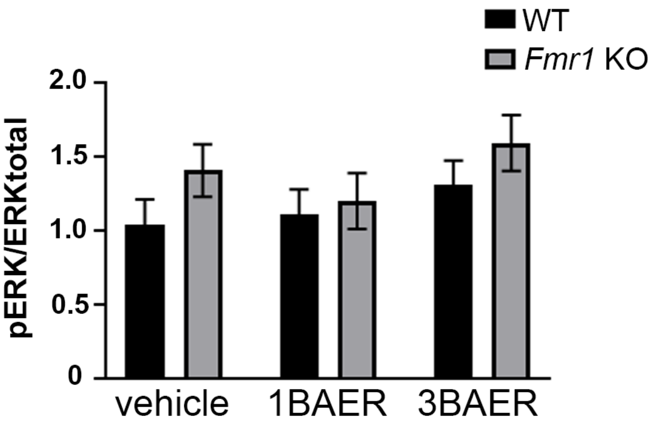

Supplementary Figure 2. ERK1/2 phosphorylation is not significantly affected by *Fmr1* genotype or BAER-101. Phosphorylated ERK1/2 and total ERK1/2 in hippocampal lysates from mice following behavioral assessment (Fig. 4 and 5) were quantified using phospho-ERK1/2- and total ERK1/2-specific ELISAs. No significant effect of genotype or treatment was detected (WT vehicle: n=10, KO veh: n=9, WT 1BAER: n=10, KO 1BAER: n=8, WT 3BAER: n=10, KO 3BAER: n=9, 2-way ANOVA, p(genotype)=0.09, p(treatment)=0.23, p(interaction)=0.73). We speculate that the handling-induced stress during behavioral assays may have affected ERK1/2 phosphorylation and masked the previously reported increase in baseline ERK1/2 phosphorylation of *Fmr1* KO hippocampus in this cohort of mice. We therefore did not assess any other molecular phenotypes.

Supplementary Table 1

Figure	Test	N	p-value	F-value	pairwise tests
**1B**	2-way ANOVA	WT veh: 12 slices<br>KO veh: 12 slices<br>WT drug: 15 slices<br>KO drug: 16 slices	p(interaction)=0.22<br>p(genotype)<0.0001<br>p(drug)=0.004	F(1,51)=1.56<br>F(1,51)=24.6<br>F(1,51)=9.1	p(wtveh/koveh)=0.0008<br>p(wtveh/kob)=0.68<br>p(koveh/kob)=0.022<br>p(wtb/kob)=0.042<br>Sidak's posthoc test
**1C**	2-way ANOVA	WT veh: 12 slices<br>KO veh: 12 slices<br>WT drug: 15 slices<br>KO drug: 16 slices	p(interaction)=0.61<br>p(genotype)=0.20<br>p(drug)=0.015	F(1,51)=0.26<br>F(1,51)=1.7<br>F(1,51)=6.3	N/A
**1D**	2-way ANOVA	WT veh: 12 slices<br>KO veh: 12 slices<br>WT drug: 15 slices<br>KO drug: 16 slices	p(interaction)=0.12<br>p(genotype)=0.95<br>p(drug)=0.71	F(1,51)=2.48<br>F(1,51)=0.005<br>F(1,51)=0.14	N/A
**1E**	Kruskal-Wallis H test	WT veh: 12<br>KO veh: 17<br>WT 1B: 10<br>KO 1B: 15<br>WT 3B: 11<br>KO 3B: 13	p=0.0001	H=24.86	p(koveh/wtveh)=0.0001<br>p(koveh/ko1B)=0.0068<br>p(koveh/ko3B)=0.0005<br>p(koveh/wt1B)=0.0003<br>p(koveh/wt3B)=0.0002<br>Wilcoxon rank sum tests with FDR-correction
**2A**	Mixed-effects model (REML)	WT veh: 4<br>KO veh: 4<br>WT 1B: 3<br>KO 1B: 6	p(day)=0.30<br><br>p(genotype)=0.007<br>p(drug)=0.93<br>p(dayXgen)=0.99<br>p(dayXdrug)=0.72<br>p(genXdrug)=0.32<br>p(dayXgenXdrug)=0.80	F (2.415, 28.07)=1.28<br>F (1, 13)=10.43<br>F (1, 13)=0.008<br>F (8, 93)=0.20<br>F (8, 93)=0.67<br>F (1, 13)=1.06<br>F (8, 93)=0.57	N/A
**2B**	2-way ANOVA	WT veh: 4<br>KO veh: 4<br>WT 1B: 3<br>KO 1B: 6	p(interaction)=0.33<br>p(genotype)=0.007<br>p(drug)=0.98	F(1,13)=0.60<br>F(1,13)=10.15<br>F(1,13)=0.0008	N/A
**2C**	2-way ANOVA	WT veh: 4<br>KO veh: 4<br>WT 1B: 3<br>KO 1B: 6	p(interaction)=0.45<br>p(genotype)=0.29<br>p(drug)=0.81	F(1,13)=1.03<br>F(1,13)=1.21<br>F(1,13)=0.06	N/A
**2D**	Mixed-effects model (REML)	WT veh: 4<br>KO veh: 4<br>WT 1B: 3<br>KO 1B: 6	p(day)=0.58<br><br>p(genotype)=0.34<br>p(drug)=0.047<br>p(dayXgen)=0.40<br>p(dayXdrug)>0.99<br>p(genXdrug)=0.52<br>p(dayXgenXdrug)=0.31	F (2.092, 24.32)=0.58<br>F (1, 13)=0.98<br>F (1, 13)=4.8<br>F (8, 93)=1.05<br>F (8, 93)=0.15<br>F (1, 13)=0.43<br>F (8, 93)=1.20	N/A
**2E**	2-way ANOVA	WT veh: 4<br>KO veh: 4<br>WT 1B: 3<br>KO 1B: 6	p(interaction)=0.50<br>p(genotype)=0.30<br>p(drug)=0.045	F(1,13)=0.48<br>F(1,13)=1.17<br>F(1,13)=4.95	N/A
**2F**	2-way ANOVA	WT veh: 4<br>KO veh: 4<br>WT 1B: 3<br>KO 1B: 6	p(interaction)=0.10<br>p(genotype)=0.87<br>p(drug)=0.046	F(1,13)=3.12<br>F(1,13)=0.03<br>F(1,13)=4.85	p(koveh/ko1b)=0.039

**3B**	2-way ANOVA	WT veh: 61 dendrites (5 mice)<br>KO veh: 94 dendrites (7 mice)<br>WT 1B: 67 dendrites (5 mice)<br>KO 1B: 78 dendrites (6 mice)	p(interaction)=0.72<br>p(treatment)=0.87<br>p(genotype)=0.0013	F(1,296)=0.13<br>F(1,296)=0.03<br>F(1,296)=10.5	N/A
**4A,B**	3-way ANOVA	WTveh: 23<br>KO veh: 21<br>WT 1B: 25<br>KO 1B: 20<br>WT 3B: 24<br>KO 3B: 20	p(interval)<0.0001<br>p(genotype)<0.0001<br>p(drug)<0.0001<br>p(intXgen)=0.53<br>p(intXdrug)<0.0001<br>p(genXdrug)=0.16<br>p(intXgenXdrug)=0.79	F (11,874)=55.8<br>F (1,216)=21.0<br>F (2,216)=34.2<br>F (11,874)=0.53<br>F (22,912)=2.65<br>F (2,216)=1.82<br>F (22,912)=0.75	p(wtveh/ko3b)=0.0015<br>FDR-corrected
**4C**	3-way ANOVA	WTveh: 23<br>KO veh: 21<br>WT 1B: 25<br>KO 1B: 20<br>WT 3B: 24<br>KO 3B: 20	p(day)<0.0001<br>p(genotype)=0.044<br>p(drug)=0.87<br>p(dayXgen)=0.70<br>p(dayXdrug)=0.33<br>p(genXdrug)=0.98<br>p(dayXgenXdrug)=0.71	F (1,86)=63.9<br>F (1,86)=4.2<br>F (2,86)=0.13<br>F (1,86)=0.15<br>F (8, 93)=0.67<br>F (2,86)=0.02<br>F (2,86)=0.34	N/A
**5A**	2-way ANOVA	WTveh: 23<br>KO veh: 19<br>WT 1B: 25<br>KO 1B: 20<br>WT 3B: 23<br>KO 3B: 20	p(interaction)=0.32<br>p(treatment)=0.13<br>p(genotype)<0.0001	F(2,81)=1.14<br>F(2,81)=0.03<br>F(1,81)=25.5	N/A
**5B**	2-way ANOVA	WTveh: 23<br>KO veh: 19<br>WT 1B: 25<br>KO 1B: 20<br>WT 3B: 23<br>KO 3B: 20	p(interaction)=0.056<br>p(treatment)=0.047<br>p(genotype)=0.0007	F(2,81)=3<br>F(2,81)=3.18<br>F(1,81)=12.42	p(wtvh/ko3b)=0.0015<br>p(kovh/ko3b)=0.019<br>FDR-corrected
**5C**	2-way ANOVA	WTveh: 23<br>KO veh: 21<br>WT 1B: 25<br>KO 1B: 20<br>WT 3B: 24<br>KO 3B: 20	p(interaction)=0.031<br>p(treatment)=0.066<br>p(genotype)=0.008	F(2,86)=3.6<br>F(2,86)=2.8<br>F(1,86)=7.5	p(wtvh/ko1b)=0.0045<br>p(wt1b/ko1b)=0.0075<br>p(ko1b/ko3b)=0.0075<br>FDR-corrected
**5D**	2-way ANOVA	WTveh: 23<br>KO veh: 21<br>WT 1B: 25<br>KO 1B: 20<br>WT 3B: 24<br>KO 3B: 20	p(interaction)=0.073<br>p(treatment)=0.62<br>p(genotype)=0.028	F(2,86)=2.7<br>F(2,86)=0.48<br>F(1,86)=5.0	p(wtvh/kovh)=0.040<br>p(kovh/ko1b)=0.51<br>p(kovh/ko3b)=0.999<br>FDR-corrected
**S1A**	Mixed-effects model (REML)	WT veh: 4<br>KO veh: 4<br>WT 1B: 3<br>KO 1B: 6	p(day)=0.16<br><br>p(genotype)=0.53<br>p(drug)=0.14<br>p(dayXgen)=0.19<br>p(dayXdrug)=0.97<br>p(genXdrug)=0.86<br>p(dayXgenXdrug)=0.64	F (1.679, 19.52)=2.02<br>F (1, 13)=0.42<br>F (1, 13)=2.54<br>F (8, 93)=1.43<br>F (8, 93)=0.28<br>F (1, 13)=0.03<br>F (8, 93)=0.75	N/A
**S1B**	2-way ANOVA	WT veh: 4<br>KO veh: 4	p(interaction)=0.86<br>p(genotype)=0.50	F(1,13)=0.03<br>F(1,13)=0.47	N/A

		WT 1B: 3<br>KO 1B: 6	p(drug)=0.14	F(1,13)=2.51	
**S1C**	2-way ANOVA	WT veh: 4<br>KO veh: 4<br>WT 1B: 3<br>KO 1B: 6	p(interaction)=0.34<br>p(genotype)=0.38<br>p(drug)=0.63	F(1,13)=0.98<br>F(1,13)=0.83<br>F(1,13)=0.25	N/A
**S1D**	Mixed-effects model (REML)	WT veh: 4<br>KO veh: 4<br>WT 1B: 3<br>KO 1B: 6	p(day)=0.14<br><br>p(genotype)=0.68<br>p(drug)=0.23<br>p(dayXgen)=0.51<br>p(dayXdrug)=0.95<br>p(genXdrug)=0.67<br>p(dayXgenXdrug)=0.51	F (1.284, 14.93)=2.34<br>F (1, 13)=0.18<br>F (1, 13)=1.55<br>F (8, 93)=0.92<br>F (8, 93)=0.34<br>F (1, 13)=0.19<br>F (8, 93)=0.91	N/A
**S1E**	2-way ANOVA	WT veh: 4<br>KO veh: 4<br>WT 1B: 3<br>KO 1B: 6	p(interaction)=0.64<br>p(genotype)=0.65<br>p(drug)=0.25	F(1,13)=0.23<br>F(1,13)=0.21<br>F(1,13)=1.46	N/A
**S1F**	2-way ANOVA	WT veh: 4<br>KO veh: 4<br>WT 1B: 3<br>KO 1B: 6	p(interaction)=0.59<br>p(genotype)=0.18<br>p(drug)=0.69	F(1,13)=0.30<br>F(1,13)=2.03<br>F(1,13)=0.17	N/A
**S1G**	Mixed-effects model (REML)	WT veh: 4<br>KO veh: 4<br>WT 1B: 3<br>KO 1B: 6	p(day)=0.16<br><br>p(genotype)=0.18<br>p(drug)=0.25<br>p(dayXgen)=0.50<br>p(dayXdrug)=0.77<br>p(genXdrug)=0.78<br>p(dayXgenXdrug)=0.88	F (2.260, 26.27)=1.95<br>F (1, 13)=2.05<br>F (1, 13)=1.48<br>F (8, 93)=0.93<br>F (8, 93)=0.61<br>F (1, 13)=0.08<br>F (8, 93)=0.47	N/A
**S1H**	2-way ANOVA	WT veh: 4<br>KO veh: 4<br>WT 1B: 3<br>KO 1B: 6	p(interaction)=0.71<br>p(genotype)=0.17<br>p(drug)=0.29	F(1,13)=0.14<br>F(1,13)=2.1<br>F(1,13)= 1.21	N/A
**S1I**	2-way ANOVA	WT veh: 4<br>KO veh: 4<br>WT 1B: 3<br>KO 1B: 6	p(interaction)=0.86<br>p(genotype)=0.46<br>p(drug)=0.21	F(1,13)=0.03<br>F(1,13)=0.58<br>F(1,13)= 1.74	N/A
**S1J**	Mixed-effects model (REML)	WT veh: 4<br>KO veh: 4<br>WT 1B: 3<br>KO 1B: 6	p(day)=0.22<br><br>p(genotype)=0.028<br>p(drug)=0.33<br>p(dayXgen)=0.68<br>p(dayXdrug)=0.55<br>p(genXdrug)=0.49<br>p(dayXgenXdrug)=0.18	F (2.526, 29.36)=1.58<br>F (1, 13)=6.1<br>F (1, 13)=1.05<br>F (8, 93)=0.72<br>F (8, 93)=0.87<br>F (1, 13)=0.51<br>F (8, 93)=1.46	N/A
**S1K**	2-way ANOVA	WT veh: 4<br>KO veh: 4<br>WT 1B: 3<br>KO 1B: 6	p(interaction)=0.42<br>p(genotype)=0.028<br>p(drug)=0.38	F(1,13)=0.71<br>F(1,13)=6.14<br>F(1,13)= 0.81	N/A
**S1L**	2-way ANOVA	WT veh: 4<br>KO veh: 4<br>WT 1B: 3<br>KO 1B: 6	p(interaction)=0.70<br>p(genotype)=0.61<br>p(drug)=0.69	F(1,13)=0.15<br>F(1,13)=0.28<br>F(1,13)= 0.17	N/A
**S2**	2-way ANOVA	WT veh: 10<br>KO veh: 9<br>WT 1B: 10	p(interaction)=0.73<br>p(genotype)=0.09<br>p(drug)=0.23	F(2,50)=0.32<br>F(1,50)=2.92<br>F(1,50)= 1.51	N/A

		KO 1B: 8<br>WT 3B: 10<br>KO 3B: 9			

Supplementary Table 1 lists statistics of all experimental data shown in the manuscript. Only results of relevant pairwise comparisons are shown. *veh=vehicle; 1B=1mg/kg BAER-101; 3B=3 mg/kg BAER-101*
